# Coronavirus Disease 2019 (COVID-19): A Literature Review from a Nursing Perspective

**DOI:** 10.37796/2211-8039.1154

**Published:** 2021-09-01

**Authors:** Amir Emami Zeydi, Mohammad Javad Ghazanfari, Farzam Shaikhi Sanandaj, Reza Panahi, Hamed Mortazavi, Keyvan Karimifar, Samad Karkhah, Joseph Osuji

**Affiliations:** 1 Department of Medical-Surgical Nursing, Nasibeh School of Nursing and Midwifery, Mazandaran University of Medical Sciences, Sari, Iran; 2 Department of Medical-Surgical Nursing, School of Nursing and Midwifery, Kashan University of Medical Sciences, Kashan, Iran; 3 Student Research Committee, School of Nursing and Midwifery, Guilan University of Medical Sciences, Rasht, Iran; 4 Geriatric Care Research Center, Department of Geriatric Nursing, School of Nursing and Midwifery, North Khorasan University of Medical Sciences, Bojnurd, Iran; 5 Student Research Committee, School of Medicine, Mashhad University of Medical Sciences, Mashhad, Iran; 6 Department of Medical-Surgical Nursing, School of Nursing and Midwifery, Guilan University of Medical Sciences, Rasht, Iran; 7 School of Nursing and Midwifery, Faculty of Health, Community, and Education, Mount Royal University, Calgary, Alberta, Canada

**Keywords:** 2019 novel coronavirus infection, COVID-19, Nursing Care, Nurse

## Abstract

**Introduction:**

As the COVID-19 pandemic ravages the world, nursing resources, and capacities play an essential role in disease management. This literature review focuses on the central issues related to the nursing care of patients affected by COVID-19.

**Material and methods:**

This literature review was conducted with an extensive search of databases, including PubMed, Web of Science (WOS), and Scopus, using the keywords “COVID19”, “2019-nCoV disease”, “2019 novel coronavirus infection”, “Nurse”, “NursingCare”, and” Nursing management.” The span of the literature search was between December 01, 2020, and January 12, 2021. A total of 28 original and English-language articles were selected for inclusion in the review.

**Results:**

Nursing interventions such as monitoring, oxygen therapy, and the use of Extra Corporeal Membrane Oxygenation (ECMO) in the care of COVID-19 patients, caring for ICU patients with COVID-19, rehabilitation of COVID-19 patients, nurses’ experiences and barriers in the care of patients with COVID-19, and also the ethical challenges in the care of patients with COVID-19, were found to be valuable in managing COVID-19 patients.

**Conclusion:**

Nurses have a pivotal role to play in the care of patients with COVID-19. Therefore, providing comprehensive and quality nursing care supported by experience and research is necessary to successfully reduce the length of hospital stay and decrease the morbidity and mortality rates of COVID-19.

## 1. Introduction

After identifying several cases of unknown pneumonia caused by a Coronavirus variant, on December 8, 2019, in Wuhan, Hubei Province, China, concerns were raised among public health professionals about the spread of a new disease in the world [1]. This particular version of the coronaviruses was not known to infect human beings in the past. However, it is now identified as the infecting organism in some patients for unknown reasons, leading to wide-spread autoimmune reactions in those affected. The virus was also implicated in human to human infections, ultimately leading to a global pandemic [2]. Epidemiological studies in COVID-19 patients have shown that the main route of transmission is from person to person through coughing or sneezing [3].

With more than 20 million nursing workforces worldwide, nurses constitute the largest proportion of health care workers (HCW) [4, 5], playing a pivotal role in the COVID-19 prevention, treatment, and rehabilitation. Considering that nurses work in different health care settings within the community, their multiple roles and responsibilities are crucial during the COVID-19 pandemic. Therefore, nurses should be well prepared to provide high quality, evidence-based, and comprehensive care for COVID-19 patients [5, 6].

Although the COVID-19 pandemic is still raging and lots of information regarding the trajectory, treatment, prevention, and control are still unfolding, it is necessary to collate some of the latest scientific information pertinent to nursing management. This literature review aims to comprehensively review the literature focusing on the central issues related to the nursing care of patients affected by COVID-19.

## 2. Material and methods

**Fig. 1:**
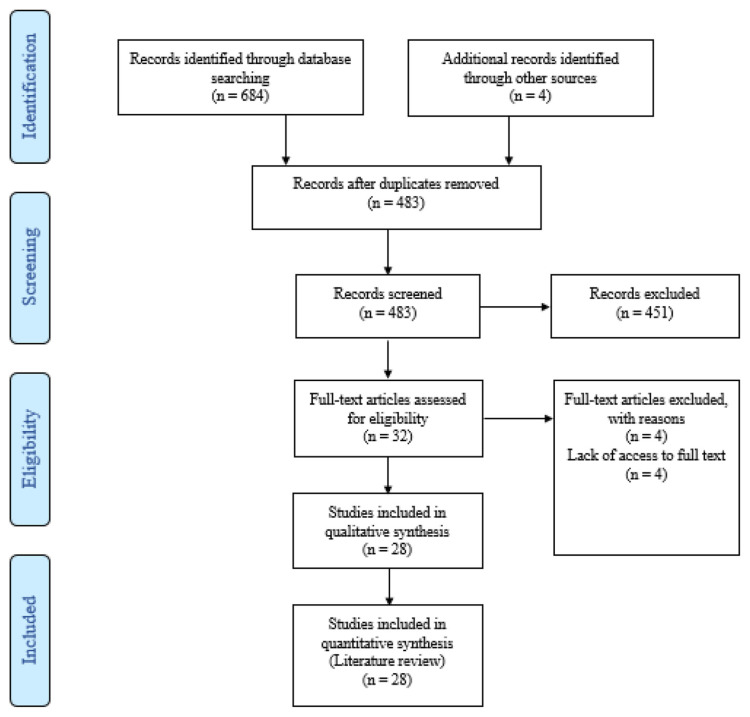
Flow diagram of study selection.

This literature review was conducted via online databases, such as PubMed, Web of Science (WOS), and Scopus from December 1, 2020, to January 12, 2021. Keywords used for the search were selected using medical subject headings (MESH) and combined with other target keywords included “COVID19”, “2019-nCoV disease”, “2019 novel coronavirus infection”, “Nurse,” “Nursing Care” and “Nursing management.” In the study, all types of English-language published articles that could potentially become useful for caring for COVID-19 patients were evaluated and included. The search was performed by two authors independently. Gray literature search was not included in the review due to the uncertainty surrounding such a novel disease condition and the exponential amount of speculation that characterized the pandemic’s early stages. To achieve maximum search comprehensiveness, lists of references from eligible studies were evaluated manually. In order to manage the data, search results were entered into the EndNote X8 software. After removing duplicate studies, the titles, abstracts, and full texts of the eligible articles were evaluated by two researchers independently. A total of 688 articles were obtained initially using database searches. Then article titles and abstracts were screened to eliminate duplicate studies, leading to the exclusion of s 656 articles. Finally, full texts of selected articles were reviewed, and 28 eligible journal articles were finally included in the review from which data were extracted for analysis (Fig. 1).

## 3. Results

### 3.1. Epidemiologic features of COVID-19

#### 3.1.1. Prevalence of COVID-19

According to the World Health Organization, a total of 88,387,352 confirmed cases of COVID-19 had been identified around the world by January 12, 2021, out of which 1,919,204 deaths have occurred. The United States with 21,761,186 infected patients (365,886 deaths), India with 10,450,284 cases (150,999 deaths), and Brazil with 8,013,708 cases (201,460 deaths), were among the countries reporting the highest numbers of cases and mortality [7].

#### 3.1.2. Clinical and epidemiologic features

Although it has been reported that one-fifth of individuals with COVID-19 remain asymptomatic [8], patients with mild infections may have nonspecific manifestations, such as fever, fatigue, cough (with or without fever), anorexia, weakness, myalgia, sore throat, shortness of breath, nasal congestion, and headache. Other uncommon symptoms such as nausea, vomiting, anosmia, dysgeusia, and diarrhea [2, 9, 10] have also been reported. In a recent meta-analysis, fever (78.8%), cough (53.9%), malaise (37.9%), and fatigue (32.2%) were listed as the most common clinical manifestations in patients with SARS-Cov2. The mean incubation period of the disease is reported to be 5.3 days [9]. Awareness of the incubation period has a useful role to play in screening and effective epidemiological control policies [11] for Covid-19.

#### 3.1.3. Modes of Disease transmission

The main route of transmission of the virus is respiratory droplets. The SARS-CoV-2 is released in the respiratory tract when an infected person coughs, sneezes, or talks. Typically, the droplets do not travel beyond 26 feet and do not remain in the air. The highest risk of transmission occurs when a patient is symptomatic, although asymptomatic transmission has been confirmed [12]. Epidemiological studies have shown that the virus is transmitted from person to person through personal contact or by touching an infected surface and then touching the nose, mouth, and eyes. Initially, the transmission of the disease through aerosols’ release was questionable [13], but currently, there is enough data to confirm the possibility of SARS_Cov2 being released by aerosol-producing procedures [14]. Although the oral-fecal method is not the primary method of transmission, it cannot be ignored because the presence of SARS-CoV-2 in the feces has been confirmed. Coronaviruses have better survivability at humidity above 30% and 25°C temperature. SARS-CoV-2 stays alive on surfaces such as metal, glass, or plastic [15] mobile phones and door handles [13] for up to 9 days. However, these surfaces can be disinfected for 1 minute using disinfection methods with 62–71% ethanol, 0.5% hydrogen peroxide, or 0.1% sodium hypochlorite. The use of hand sanitizers and the disinfection of the environment and patient care equipment are essential infection prevention and control strategies, both within the hospital and in community settings [2].

#### 3.1.4. Age-dependent effects of COVID-19

The age distribution of affected hospitalized patients is mostly middle-aged people (people older than 30 years) and older adults. Morbidity and mortality rates are highest among older adult patients hospitalized in the intensive care unit (ICU). The clinical manifestations in these patients progress more rapidly and often lead to severe respiratory failure (9). Also, it has been reported that up to 50.9% of COVID-19 patients had underlying diseases [16]. Evidence suggests that infection is rare in children and is usually mild, and when children are infected, about 18% of cases remain asymptomatic. The most common symptoms of COVID-19 in children have been reported as fever (51.2%) and cough (37%) [17].

#### 3.1.5. Risk Factors for COVID-19 induced ARDS and Progression to Death

As the prevalence and spread of COVID-19 increases worldwide, many more deaths are likely to be recorded. Older adults and people with underlying diseases, such as respiratory and cardiovascular diseases, are at higher risk. Smoking and obesity are associated with an increased risk of death [18]. In Italy, the risk of death and disease severity was higher in smokers and men than in women [19]. A Recently published meta-analysis reveals that chronic respiratory diseases, hypertension, cardiovascular disease, chronic kidney disease, cerebrovascular disease, malignancy, diabetes, and obesity are most typical risk factors COVID-19 severity [20]. [20]The most common complication in patients with COVID-19, which causes high mortality, is acute respiratory distress syndrome (ARDS) [21]. In one study, 45% of people who died from COVID-19 were as a result of ARDS development [22]. The most common symptom of COVID-19 patients with ARDS is shortness of breath. The risk factors associated with the development of ARDS and progression from ARDS to death include older age (>65 years), neutrophilia, organ and coagulation dysfunction, and higher lactate dehydrogenase and D-dimer [23]. Vitamin D deficiency has been proposed as a risk factor for ARDS development in CIVID-19 patients. Vitamin D deficiency causes more cellular inflammation and cytokine release within 48 hours of the development of ARDS. Also, a deficiency of thiamine and selenium increases the risk of developing ARDS [24, 25]. Despite all the risks factors mentioned above, the Italian healthcare system’s experience revealed that the increase in the nurses’ workload and the shortage of beds during the COVID-19 pandemic increased the mortality rate of the disease dramatically, underscoring the need to train more HCW and provide adequate care infrastructure in order to reduce morbidity and mortality of COVID-19 [26].

#### 3.1.6. Infection prevention measures in the hospital setting

Today, millions of HCW, especially nurses, are in the front-line of the global battle to treat COVID-19 patients and flatten the curve of transmission. Reports from China show that about 3,300 HCW were infected by February 2020, with at least 22 of them dying by the end of March [27]. The results of a recently published systematic review regarding the global prevalence of infection and mortality from COVID-19 among HCW showed that a total of 152 888 infections and 1413 deaths were reported among HCWs during the early phases of the pandemic [28]. The high number of cases and deaths among HCW resulted in a severe shortage of staff, and extreme fatigue and stress among nurses, with the likelihood of weakening the immune system and subsequent increase in infection rates [29]. With the increasing prevalence of this disease, the lack of personal protective equipment (PPE) had become a significant concern for health care providers, as it is the first step in protecting them [30]. The availability and use of appropriate personal protective equipment (PPE) such as face masks, eye protectors, protective clothing, and body coverings, including shoes and safety goggles [31, 2], are effective strategies used to prevent the spread of infections among HCW. To wear protective cover, nurses need to tie their hair, hold it in place, and remove watches and jewellery during patient care to prevent contamination. To prevent dehydration, it is essential for nurses to drink water before wearing PPE and use the bathroom as necessary. In the event of any contamination, damage, or rupture of full-body clothing, the PPE must be replaced. Nurses should also replace gloves when they get wet [31].

It is best to use N95 masks or surgical masks during patient care procedures [32]. It has been previously shown that the incidence rate of respiratory infections in HCW who wore a surgical mask was twice as high as those who wore N95 masks [33, 34]. Comparatively, medical masks and N95 masks did not differ in protecting HCW during non–aerosol care. However, N95 should be used during short-term aerosol-generating procedures and high-risk care [35].

Given that protection against aerosols’ larger than 0.3 microns entry into N95 masks is unknown, research data suggests that N95 masks are more effective than surgical masks in preventing the spread of infections, but this has not been definitively clarified [36]. Patients whose care increases aerosol generation’s possibility should be placed in isolation units, with all precautions taken to prevent infecting those who care for them, especially nurses [14]. Nurses should avoid being infected by contaminated secretions of patients during interventions such as assisting patients with a nebulizer, chest physiotherapy, bronchoscopy, tracheostomy, intubation, orotracheal suction, manual ventilation before intubation, non-invasive ventilation, cardiopulmonary resuscitation, gastroscopy, and collection of laboratory samples [14, 37]. Nurses should use PPE when collecting samples from COVID-19 patients or suspected patients, and then the samples should be sent separately in non-perforated bags, along with the laboratory requisition forms [37]. Preferably, immediately after the end of each day, all equipment, floors, nursing stations, and other areas of hospital wards must be disinfected with 2 or 3% hydrogen peroxide [38].

### 3.2. Nursing Care of Patients with COVID-19

#### 3.2.1. Monitoring

Precise and continuous monitoring of COVID-19 patients by nurses is crucial for recognizing patients’ deterioration and occurrence of any potential complications. The patient’s vital signs should be monitored continuously, with particular attention paid to respiration rate, oxygen saturation (SPO2), and changes in consciousness level. Clinically, common and significant symptoms of COVID-19 such as cough, shortness of breath, fever, sputum, and chest tightness, should be monitored. If needed, the patient’s arterial blood gas (ABG) results should be evaluated at frequent intervals [39]. Patients’ temperature should be checked, and any temperature greater than 37.3°C should be reported and followed up. Surgical masks should be offered immediately to patients with symptoms of cough and sneezing. Liquids or IV fluid therapy should be used to prevent dehydration and worsening respiratory status when appropriate. As a result of the anorexia experienced by these patients and the need to strengthen their immunity, their diet should contain a balanced ratio of protein, carbohydrates, vitamins, and minerals [40]. Although continuous monitoring of the ‘ patients respiratory system is vital, nurses should not neglect other organs’ or forget to assess other body systems.

#### 3.2.2. Oxygen therapy and ECMO in care of COVID-19 patients

Acute hypoxemic respiratory failure or ARDS is the most common and severe complication of COVID-19, which requires oxygen and ventilation therapy. For patients with mild or moderate respiratory problems and hypoxemia, oxygen therapy using a nasal cannula, simple face mask, or reservoir mask may be enough. The appropriate flow rate of oxygen should be determined based on the patient’s condition. If oxygen therapy does not achieve the oxygen saturation target range, nurses should investigate the potential causes and use other oxygen delivery devices or methods [41, 2]. Extracorporeal Membrane Oxygenation (ECMO) may be an effective modality for COVID-19-related acute hypoxemic respiratory failure [42]. Given the effectiveness of ECMO in Middle East Respiratory Syndrome Coronavirus (MERS-COV) patients with ARDS, this method appears to be effective in COVID-19, but the data are inconsistent. Contrary to the recommendations for using ECMO in COVID-19 patients with ARDS [43], this treatment was not effective in some cases [44]. Care of patients receiving oxygen therapy using different devices is briefly shown in Table 1.

#### 3.2.3. Caring for ICU patients with COVID-19

In the ICU, two nurses per patient must perform proper safe care and isolation of patients with COVID-19 [37]. In COVID-19 patients with ARDS, a high-flow nasal cannula (HFNC) and non-invasive ventilation (NIV) are useful in maintaining positive end-expiratory pressure (PEEP) and preventing alveolar collapse. Prone positioning combined with low tidal volume (6 ml/kg of ideal body weight) and neuromuscular blocking agents improves ARDS patients’ oxygen therapy [45, 46]. A systematic review revealed prone positioning could significantly improve the oxygenation and perfusion in COVID-19 patients [47]. Besides, prone positioning is recommended for oxygen improvement in mechanically ventilated COVID-19 patients with severe ARDS [48]. Patients who are ventilated and placed in the prone position are at risk for complications such as endotracheal tube displacement, limited access to the venous route, bruising around the mouth, bedsores, possible kinking of catheters, periorbital and facial edema, increased oral secretions, and skin damage [46]. In patients predisposed to pressure injuries, nurses must perform continuous risk assessments and change patients’ positions at regular intervals. [49]. Simultaneously, the nurse should examine the patient for bed sores and prevent falls, tube slipping, and eye damage caused by pressure, skin and mouth damage, and other complications [2]. A major limitation in patients in the prone position is a potential difficulty encountered if the patient requires to be intubated and the requirement for 3 to 5 people to participate in the procedure [50]. Restriction of fluid intake is effective in relieving pulmonary edema [43]. If the patient has ARDS or has the necessary criteria, the patient may require invasive mechanical ventilation. [2] Although there is no single ‘Silver Bullet’ to cure COVID-19, a clinical expert panel called the frontline COVID-19 Critical Care Alliance proposed a promising management protocol (MATH+: a combination of intravenous methylprednisolone, high dose intravenous ascorbic acid, thiamine, full anticoagulation with heparin and other co-interventions) as a life-saving approach in critically ill or other COVID-19 patients [51, 52]. However, this treatment protocol should be investigated and confirmed in future studies in critical care units.

The use of a closed airway suction device, convalescent plasma therapy for severe and critically ill patients, the use of lung-protective strategy in patients with ARDS, avoidance of excessive PEEP, fluid resuscitation with crystalloids, monitoring of signs of secondary infection in patients admitted to ICU> 48 h, and early nutrition therapy during 24–48 hours after admission are other recommended interventions in ICU COVID-19 patients [53].

#### 3.2.4. Other Nursing Care and rehabilitation of COVID-19 patients

As the number of new COVID-19 infections is increasing daily, nurses must isolate patients and prevent virus spread when transferring these patients between wards. Nurses must consider five principles when transferring patients; these include recognizing patients in the acute phase of the disease, the nurses’ safety, protecting others, availability of emergency treatment measures, and the possibility of infecting others after the patient’s transfer is completed. During patient transfer, a physician or nurse who can manage emergency conditions should accompany the patient. The patient needs constant monitoring of blood pressure, pulse, pulse oximetry, and CO2 levels and may require a defibrillator. Nurses must wear N95 masks and PPE to ensure their safety, and the patients must wear surgical masks, if possible. During patient transport, a specific route must be planned, and after the transfer, the route must be disinfected and the nurses’ protective clothing replaced [54].

With the increasing spread of the COVID-19 pandemic and the high number of patients admitted to the ICU, patients who survive and are discharged from the ICU need rehabilitation. Rehabilitation is a vital part of patient-centered care in response to the COVID-19 crises and plays an essential role in accelerating recovery after discharge from the ICU [55]. These patients may develop post-ICU syndrome and complications that may include immobility, venous thromboembolism, delirium [56], depression [57], post-traumatic stress disorder (PTSD) [58], and anxiety [59]. Rehabilitation should be done in short-term, medium, and long-term programs for patients and their families. Maintaining an active relationship between nurses and patients’ families plays an essential role in providing adequate care during the rehabilitation process. After discharge from the ICU, patients may be negatively impacted by prolonged use of sedatives, immobility, mechanical ventilation, and delirium, and may depend on personal and daily care from HCW. Many surviving COVID-19 patients need to be admitted to a rehabilitation centre to improve their functioning and be prepared to re-enter society [55].

### 3.3. Nurses’ experiences in the Care of Patients with COVID-19

Paying attention to nurses’ experiences in the COVID-19 pandemic can provide the best foundation for better crisis management in the future. Nurses’ experiences during the Covid-19 Pandemic can be evaluated in various positive and negative dimensions. Nurses’ negative experiences were usually related to difficulties in coping with increased work and family demands during the pandemic. As the pandemic ravages the world, management issues were among the most critical negative experiences of nurses. These include nursing staff shortages, long shifts, scarcity of resources, and PPE shortages [60, 61]. Long shifts in the hospital, being away from family, and end-of-life care can cause emotional problems among nurses and other HCW [62, 61]. In two qualitative studies from Turkey [63] and Iran [64], nurses experienced various psychological distress during the care of COVID-19 patients.

In contrast, nurses’ positive experiences resulted from the nursing profession’s positive contributions during the COVID-19 pandemic. Nurses’ spoke of positive comments and more respect from other people for their sacrifices. This pandemic has led to a better appreciation of the nursing profession’s contributions in managing complex public health problems and patient recovery. These positive developments have provided new opportunities for developing the nursing profession [61]. Strengthening the spirit of cooperation, pride in oneself as a committed nurse, strengthening self-confidence in caring for patients, and public support for nurses and other HCW, have been other positive experiences of nurses during the COVID-19 pandemic [60].

### 3.4. Nursing management during the COVID-19 Pandemic

As the COVID-19 crisis continues, nurse managers must make quick, creative, practical, and useful decisions and use appropriate methods to engage patients and their families during the disease process. Nursing personnel administrators must provide the appropriate emotional and physical support for nurses to enable them to meet the increased expectations placed on them [65]. Nurse managers should also develop management plans to provide high quality, safe, and cost-effective care [66]. The main aspects in human resource management during this crisis include: clarifying human resource structures, standardizing communication procedures, securing an adequate number of HCW and other staff, restricting high-risk procedures/behaviours, and developing flexible shifts for nurses [67]. Creating positive interactions between patients, families, and nurses to provide care can play a vital role in managing COVID-19 [65]. Appropriate instruction and interaction after discharge to provide relevant patients’ care needs such as medication adherence, diet, psychological counseling, and observance of care standards are necessary and can be achieved by Tele-nursing. Tele-nursing improves the quality of care and treatment outcomes, controls treatment costs, reduces the need for emergency room visits, and encourages patient and family involvement in care decisions to achieve a high self-management level [68].

### 3.5. Nurses’ barriers to caring for patients with COVID-19

There are several barriers for nurses in caring for COVID-19 patients. Most important of these are limited and ambiguous information about COVID-19 and inadequate support for HCW, such as a lack of facilities and PPE. Also, concerns about their family safety and emotional and psychological stress were other barriers reported by nurses as they cared for COVID-19 patients [69].

### 3.6. Ethical Challenges in the Care of Patients with COVID-19

With the spread of COVID-19, many patients are admitted into hospitals for care. Lack of sufficient medical equipment, PPE, nursing and medical staff shortages, and bedding have created ethical challenges for nurses and other HCW [70]. Due to insufficient and inadequate medical resources, medical staff had to impose restrictions on Italy’s patient care [70]. For example, younger people may be preferred for admission into ICU and mechanical ventilation [71] over older patients. In the United States, do-not-attempt-resuscitate (DNAR) has been recommended for some patients due to a shortage of respiratory support devices, ICU spaces, and PPE [72]. Another challenge in patients with COVID-19 is cardiopulmonary resuscitation (CPR) [73]. It may take up to 10 minutes to enter the patient’s room, remove the clothing, and prepare to resuscitate the patient. The same delay in CPR reduces the possibility of survival of a patient with COVID-19 by up to 10%. The lack of sufficient equipment and medical personnel, the implementation or non-implementation of CPR on patients over 80 who have undergone cardiac arrest are considered some of the major ethical challenges [71] encountered during the care of COVID-19 patients. Therefore, resource allocation during a pandemic should be based on the maximum use of limited resources, the equitable treatment of different individuals, and resource prioritization of care according to how critical the patients’ needs are [74].

## 4. Conclusion

The global prevalence of COVID-19 requires nurses’ active participation as the most extensive and primary professionals at the forefront of the fight against the pandemic. The fight against COVID-19 requires a combination of care based on scientific evidence, education and information sharing, public health, and sound policy. Nurses have a pivotal role in caring for patients with COVID-19. Providing comprehensive nursing care of the highest quality, supported by experience and research, can successfully reduce patients’ length of hospital stay, reduce morbidity and mortality rates of the disease, and promote patients’ recovery rate. As the COVID-19 crisis rages on, nurse managers should also develop management plans to provide high-quality, safe, and cost-effective care to patients while ensuring that nursing staff is protected while caring for patients.

## Figures and Tables

**Table 1:** Oxygen therapy in the care of COVID-19 patients.

Oxygen therapy methods	Rate	FiO_2_	Nursing Care
Low Flow Oxygen Delivery Devices			Provide accurate information on oxygen delivery devices to engage the patient before oxygen therapy. Use a low dose sedative. Use a proper nasal catheter based on the diameter of the patient’s nasal cavity. Use decompression plaster to prevent facial skin damage and head strap tightness. Adjust the water level in the humidifier chamber. Control flow rate, FiO2, and water temperature based on the patient’s respiratory needs. Record and report the following to the doctor: hemodynamic instability, respiratory distress, hypoxemia persists despite oxygen therapy, deterioration of consciousness, breathing rate more than 40 cycles per minute, significant sputum [75].
Nasal cannula	1–6 liter/min	24–44%	
Simple (Hudson) Mask	5–10 liter/min	35–60%	
Venturi mask	2–15 liter/min	24–60%	
Reservoir bag (non-rebreather mask)	15 liter/min	85–90%	
High Flow Oxygen Delivery Devices			✓ Ordinary oxygen therapy devices that carry dry oxygen cause mask discomfort, dry nose and mouth, eye irritation, nasal and eye damage, bloating, and aspiration. Adequate moisture supply through HFNC can prevent airway dryness and inflammatory response due to mucosal dryness [75]. ✓ Patients’ sputum and secretions should be cleaned using tissue paper and disposed of in a chlorine-containing disinfectant (2500 mg/L). ✓ Patients’ secretions can be disposed of using an oral mucus extractor or suctioning tube in a chlorine-containing disinfectant collector (2500 mg/L) [39].
Facial mask with Venturi valve	Oxygen flow rate does not depend on minutes.	24–30%	
High Flow Nasal Cannula	1–60 liter/min	24–70%	
Non-Invasive Ventilation			Patients who receive CPAP/BiPAP through the nose may complain of nasal and pharyngeal discomfort. Therefore, it is possible to reduce the side effects of ventilation by adding sufficient warm humidity. Low ambient temperature and humidity in the circuit may cause water to accumulate in the nostrils and interfere with the patient’s sleep, so nurses should consider factors affecting humidity, such as the patient’s breathing pattern, the flow delivered to the HFNC, and note the type of device and the appropriate position of the nasal cannula [76].
Continuous positive airway pressure (CPAP) Bilevel positive airway pressure (BiPAP)			
Extracorporeal membrane oxygenation (ECMO)			✓ Nurses need to quickly diagnose any neurological changes, such as: increase in the size of the pupil, change in skin color and temperature, decrease in oxygen saturation, hypotension or hypertension, and take appropriate actions. ✓ Nurses should examine patients under ECMO for colds, blemishes, and pale skin. ✓ If there is any ischemia, these patients pulses must be monitored every 4 hours or less and, inform the doctor immediately and take the necessary measures [77, 2]. ✓ Nurses should evaluate COVID-19 patients on ECMO for erythema, pus, integrity and dressing efficiency, canula constancy, ecchymosis in the abdomen and inguinal areas, hypotension, and acute anemia. ✓ The patients’ body temperature should be assessed for fever for early diagnosis of infection. ✓ The use of steroids in ARDS and adrenal insufficiency, body temperature control, and blood transfusion following cardiac surgery can be effective in reducing the risk of infection in patients under ECMO [78]. ✓ Patients with COVID-19 who are in ICU and are supported by ECMO are at risk for bed sores due to hemodynamic instability and high doses of multiple vasopressors, which lead to decreased tissue perfusion. Therefore, it is necessary to provide special skin care in patients with COVID-19 who are supported by ECMO [78].

## References

[1] Karkhah S Ghazanfari MJ Shamshirian A Panahi L Molai M Zeydi AE Clinical Features of Patients with Probable 2019 Novel Coronavirus Infected Pneumonia in Rasht, Iran: A Retrospective Case Series Open Access Maced J Med Sci 2020 8 T1 16 22

[2] Jin Y-H Cai L Cheng Z-S Cheng H Deng T Fan Y-P A rapid advice guideline for the diagnosis and treatment of 2019 novel coronavirus (2019-nCoV) infected pneumonia (standard version) Mil Med Res 2020 7 1 4 32029004 10.1186/s40779-020-0233-6 PMC7003341

[3] Rothan HA Byrareddy SN The epidemiology and pathogenesis of coronavirus disease (COVID-19) outbreak J Autoimmun 2020 109 102433 32113704 10.1016/j.jaut.2020.102433 PMC7127067

[4] Ahanchian MR Zeydi AE Armat MR Conflict management styles among Iranian critical care nursing staff: a cross-sectional study Dimens Crit Care Nurs 2015 34 3 140 5 25840129 10.1097/DCC.0000000000000106

[5] Karimi Moonaghi H Emami Zeydi A Mirhaghi A Patient education among nurses: bringing evidence into clinical applicability in Iran Invest Educ Enferm 2016 34 1 137 51 28569983 10.17533/udea.iee.v34n1a16

[6] Choi KR Skrine Jeffers K Cynthia Logsdon M Nursing and the novel coronavirus: Risks and responsibilities in a global outbreak J Adv Nurs 2020 76 7 1486 7 32202336 10.1111/jan.14369 PMC7228354

[7] Organization WH Coronavirus disease 2019 (COVID-19): situation report [Internet] 2021 [cited 2021 January 12]. Available from, https://www.who.int/publications/m/item/weekly-epidemiological-update-12-january-2021

[8] Kim G-u Kim M-J Ra SH Lee J Bae S Jung J Clinical characteristics of asymptomatic and symptomatic patients with mild COVID-19 Clin Microbiol Infect 2020 26 7 948.e1 e3 32360780 10.1016/j.cmi.2020.04.040 PMC7252018

[9] Liu M He P Liu H Wang X Li F Chen S Clinical characteristics of 30 medical workers infected with new coronavirus pneumonia Zhonghua Jie He He Hu Xi Za Zhi 2020 43 E016. 0 32062957 10.3760/cma.j.issn.1001-0939.2020.0016

[10] Zhao S Zhuang Z Cao P Ran J Gao D Lou Y Quantifying the association between domestic travel and the exportation of novel coronavirus (2019-nCoV) cases from Wuhan, China in 2020: a correlational analysis J Travel Med 2020 27 2 taaa022 32080723 10.1093/jtm/taaa022 PMC7107546

[11] Jiang X Rayner S Luo MH Does SARS-CoV-2 has a longer incubation period than SARS and MERS? J Med Virol 2020 92 5 476 8 32056235 10.1002/jmv.25708 PMC7166592

[12] Wimalawansa SJ Global Epidemic Of Coronavirus—Covid-19: What Can We Do To Minimize Risks Eur J Biomed Pharm Sci 2020 7 3 432 8

[13] Wang L Shi Y Xiao T Fu J Feng X Mu D Chinese expert consensus on the perinatal and neonatal management for the prevention and control of the 2019 novel coronavirus infection Ann Transl Med 2020 8 3 47 32154287 10.21037/atm.2020.02.20 PMC7036629

[14] Brosseau LM Are powered air purifying respirators a solution for protecting healthcare workers from emerging aerosol-transmissible diseases? Ann Work Expo Health 2020 64 4 339 41 32154831 10.1093/annweh/wxaa024

[15] Kampf G Todt D Pfaender S Steinmann E Persistence of coronaviruses on inanimate surfaces and their inactivation with biocidal agents J Hosp Infect 2020 104 3 246 51 32035997 10.1016/j.jhin.2020.01.022 PMC7132493

[16] Xiong S Liu L Lin F Shi J Han L Liu H Clinical characteristics of 116 hospitalized patients with COVID-19 in Wuhan, China: a single-centered, retrospective, observational study BMC Infect Dis 2020 20 1 787 33092539 10.1186/s12879-020-05452-2 PMC7578439

[17] Ding Y Yan H Guo W Clinical characteristics of children with COVID-19: a meta-analysis Front Pediatr 2020 8 431 32719759 10.3389/fped.2020.00431 PMC7350605

[18] Wang T Du Z Zhu F Cao Z An Y Gao Y Comorbidities and multi-organ injuries in the treatment of COVID-19 Lancet 2020 395 10228 e52 32171074 10.1016/S0140-6736(20)30558-4 PMC7270177

[19] Jordan RE Adab P Cheng K Covid-19: risk factors for severe disease and death BMJ 2020 368 m1198 32217618 10.1136/bmj.m1198

[20] Zhou Y Yang Q Chi J Dong B Lv W Shen L Comorbidities and the risk of severe or fatal outcomes associated with coronavirus disease 2019: A systematic review and meta-analysis Int J Infect Dis 2020 99 47 56 32721533 10.1016/j.ijid.2020.07.029 PMC7381888

[21] Jamaati H Dastan F Tabarsi P Marjani M Saffaei A Hashemian SM A Fourteen-day Experience with Coronavirus Disease 2019 (COVID-19) Induced Acute Respiratory Distress Syndrome (ARDS): An Iranian Treatment Protocol Iran J Pharm Sci 2020 19 1 31 6 10.22037/ijpr.2020.113337.14239 PMC7462513 32922466

[22] Huang C Wang Y Li X Ren L Zhao J Hu Y Clinical features of patients infected with 2019 novel coronavirus in Wuhan, China Lancet 2020 395 10223 497 506 31986264 10.1016/S0140-6736(20)30183-5 PMC7159299

[23] Wu C Chen X Cai Y Zhou X Xu S Huang H Risk factors associated with acute respiratory distress syndrome and death in patients with coronavirus disease 2019 pneumonia in Wuhan, China JAMA Intern Med 2020 180 7 934 43 32167524 10.1001/jamainternmed.2020.0994 PMC7070509

[24] Jain A Chaurasia R Sengar NS Singh M Mahor S Narain S Analysis of vitamin D level among asymptomatic and critically ill COVID-19 patients and its correlation with inflammatory markers Sci Rep 2020 10 1 1 8 33214648 10.1038/s41598-020-77093-z PMC7677378

[25] Mardani R Alamdary A Nasab SM Gholami R Ahmadi N Gholami A Association of vitamin D with the modulation of the disease severity in COVID-19 Virus Res 2020 289 198148 32866536 10.1016/j.virusres.2020.198148 PMC7455115

[26] Lucchini A Giani M Elli S Villa S Rona R Foti G Nursing Activities Score is increased in COVID-19 patients Intensive Crit Care Nurs 2020 59 102876 32360493 10.1016/j.iccn.2020.102876 PMC7177066

[27] Lancet T COVID-19: protecting health-care workers Lancet (London, England) 2020 395 10228 922 10.1016/S0140-6736(20)30644-9 PMC7138074 32199474

[28] Bandyopadhyay S Baticulon RE Kadhum M Alser M Ojuka DK Badereddin Y Infection and mortality of healthcare workers worldwide from COVID-19: a systematic review BMJ Glob Health 2020 5 12 e003097 10.1136/bmjgh-2020-003097 PMC7722361 33277297

[29] Lai J Ma S Wang Y Cai Z Hu J Wei N Factors associated with mental health outcomes among health care workers exposed to coronavirus disease 2019 JAMA Netw Open 2020 3 3 e203976 e76 32202646 10.1001/jamanetworkopen.2020.3976 PMC7090843

[30] Zhou P Huang Z Xiao Y Huang X Fan X-G Protecting Chinese healthcare workers while combating the 2019 novel coronavirus Infect Control Hosp Epidemiol 2020 41 6 745 6 32131906 10.1017/ice.2020.60 PMC7184141

[31] Huh S How to train the health personnel for protecting themselves from novel coronavirus (COVID-19) infection during their patient or suspected case care J Educ Eval Health Prof 2020 17 10 32150796 10.3352/jeehp.2020.17.10 PMC7162995

[32] Long Y Hu T Liu L Chen R Guo Q Yang L Effectiveness of N95 respirators versus surgical masks against influenza: a systematic review and meta-analysis J Evid Based Med Healthc 2020 13 2 93 101 10.1111/jebm.12381 PMC7228345 32167245

[33] MacIntyre CR Wang Q Cauchemez S Seale H Dwyer DE Yang P A cluster randomized clinical trial comparing fit-tested and non-fit-tested N95 respirators to medical masks to prevent respiratory virus infection in health care workers Influenza Other Respir Viruses 2011 5 3 170 9 21477136 10.1111/j.1750-2659.2011.00198.x PMC4941587

[34] Radonovich LJ Simberkoff MS Bessesen MT Brown AC Cummings DA Gaydos CA N95 respirators vs medical masks for preventing influenza among health care personnel: a randomized clinical trial Jama 2019 322 9 824 33 31479137 10.1001/jama.2019.11645 PMC6724169

[35] Bartoszko JJ Farooqi MAM Alhazzani W Loeb M Medical masks vs N95 respirators for preventing COVID-19 in healthcare workers: A systematic review and meta-analysis of randomized trials Influenza Other Respir Viruses 2020 10.1111/irv.12745 PMC7298295 32246890

[36] Smith JD MacDougall CC Johnstone J Copes RA Schwartz B Garber GE Effectiveness of N95 respirators versus surgical masks in protecting health care workers from acute respiratory infection: a systematic review and meta-analysis Cmaj 2016 188 8 567 74 26952529 10.1503/cmaj.150835 PMC4868605

[37] Jansson M Liao X Rello J Strengthening ICU health security for a coronavirus epidemic Intensive Crit Care Nurs 2020 57 102812 32044122 10.1016/j.iccn.2020.102812 PMC7135420

[38] New Recommendations P A for Health Care Providers: Coronavirus Disease 2019 (COVID-19) 2020 [Internet].

[39] Liang T Handbook of COVID-19 prevention and treatment The First Affiliated Hospital 68 Zhejiang University School of Medicine Compiled According to Clinical Experience 2020

[40] Malhotra N Joshi M Datta R Bajwa SJS Mehdiratta L Indian society of anaesthesiologists (ISA national) advisory and position statement regarding COVID-19 Indian J Anaesth 2020 64 4 259 32362681 10.4103/ija.IJA_288_20 PMC7189907

[41] O’Driscoll B Howard L Earis J Mak V British Thoracic Society Guideline for oxygen use in adults in healthcare and emergency settings BMJ Open Respir Res 2017 4 1 e000170 10.1136/bmjresp-2016-000170 PMC5531304 28883921

[42] Barbaro RP MacLaren G Boonstra PS Iwashyna TJ Slutsky AS Fan E Extracorporeal membrane oxygenation support in COVID-19: an international cohort study of the Extracorporeal Life Support Organization registry Lancet 2020 396 10257 1071 8 32987008 10.1016/S0140-6736(20)32008-0 PMC7518880

[43] Sun Q Qiu H Huang M Yang Y Lower mortality of COVID-19 by early recognition and intervention: experience from Jiangsu Province Ann Intensive Care 2020 10 1 1 4 32189136 10.1186/s13613-020-00650-2 PMC7080931

[44] Henry BM Lippi G Poor survival with extracorporeal membrane oxygenation in acute respiratory distress syndrome (ARDS) due to coronavirus disease 2019 (COVID-19): Pooled analysis of early reports J Crit Care 2020 58 27 8 32279018 10.1016/j.jcrc.2020.03.011 PMC7118619

[45] Guérin C Reignier J Richard J-C Beuret P Gacouin A Boulain T Prone positioning in severe acute respiratory distress syndrome N Engl J Med 2013 368 23 2159 68 23688302 10.1056/NEJMoa1214103

[46] Guérin C Prone positioning acute respiratory distress syndrome patients Ann Transl Med 2017 5 14 289 28828364 10.21037/atm.2017.06.63 PMC5537107

[47] Mch SA Baishya M Singh A Khanna P Effect of awake prone positioning in COVID-19 patients-A systematic review Trends Anaesth Crit Care 2021 36 17 22 10.1016/j.tacc.2020.09.008 PMC7521914 38620706

[48] Weiss TT Cerda F Scott JB Kaur R Sungurlu S Mirza SH Prone positioning for patients intubated for severe acute respiratory distress syndrome (ARDS) secondary to COVID-19: a retrospective observational cohort study Br J Anaesth 2021 126 1 48 55 33158500 10.1016/j.bja.2020.09.042 PMC7547633

[49] Boyko TV Longaker MT Yang GP Review of the current management of pressure ulcers Adv Wound Care 2018 7 2 57 67 10.1089/wound.2016.0697 PMC5792240 29392094

[50] Ghelichkhani P Esmaeili M Prone position in management of COVID-19 patients; a commentary Arch Acad Emerg Med 2020 8 1 e48 32309812 PMC7158870

[51] Kory P Meduri GU Iglesias J Varon J Marik PE Clinical and Scientific Rationale for the “MATH+” Hospital Treatment Protocol for COVID-19 J Intensive Care Med 2020 36 2 135 56 33317385 10.1177/0885066620973585

[52] Marik PE Kory P Varon J Iglesias J Meduri GU MATH+ protocol for the treatment of SARS-CoV-2 infection: the scientific rationale Expert Rev Anti Infect Ther 2020 1 7 10.1080/14787210.2020.1808462 32809870

[53] Shang Y Pan C Yang X Zhong M Shang X Wu Z Management of critically ill patients with COVID-19 in ICU: statement from front-line intensive care experts in Wuhan, China Ann Intensive Care 2020 10 1 1 24 32506258 10.1186/s13613-020-00689-1 PMC7275657

[54] Liew MF Siow WT Yau YW See KC Safe patient transport for COVID-19 Crit Care 2020 24 1 1 3 32183864 10.1186/s13054-020-2828-4 PMC7079436

[55] Simpson R Robinson L Rehabilitation After Critical Illness in People With COVID-19 Infection Am J Phys Med Rehabil 2020 99 6 470 32282359 10.1097/PHM.0000000000001443 PMC7253039

[56] Kotfis K Marra A Ely EW ICU delirium—A diagnostic and therapeutic challenge in the intensive care unit Anaesthesiol Intensive Ther 2018 50 2 128 40 29882581 10.5603/AIT.a2018.0011

[57] Rabiee A Nikayin S Hashem MD Huang M Dinglas VD Bienvenu OJ Depressive symptoms after critical illness: a systematic review and meta-analysis Crit Care Med 2016 44 9 1744 53 27153046 10.1097/CCM.0000000000001811 PMC7418220

[58] Parker AM Sricharoenchai T Raparla S Schneck KW Bienvenu OJ Needham DM Posttraumatic stress disorder in critical illness survivors: a metaanalysis Crit Care Med 2015 43 5 1121 9 25654178 10.1097/CCM.0000000000000882

[59] Nikayin S Rabiee A Hashem MD Huang M Bienvenu OJ Turnbull AE Anxiety symptoms in survivors of critical illness: a systematic review and meta-analysis Gen Hosp Psychiatry 2016 43 23 9 27796253 10.1016/j.genhosppsych.2016.08.005 PMC5289740

[60] Lee N Lee H-J South Korean Nurses’ Experiences with Patient Care at a COVID-19-Designated Hospital: Growth after the Frontline Battle against an Infectious Disease Pandemic Int J Environ Res Public Health 2020 17 23 9015 10.3390/ijerph17239015 PMC7729510 33287343

[61] Galehdar N Toulabi T Kamran A Heydari H Exploring nurses’ perception of taking care of patients with coronavirus disease (COVID-19): A qualitative study Nurs Open 2021 8 1 171 9 33318825 10.1002/nop2.616 PMC7729793

[62] Karimi Z Fereidouni Z Behnammoghadam M Alimohammadi N Mousavizadeh A Salehi T The lived experience of nurses caring for patients with COVID-19 in Iran: a phenomenological study Risk Manag Healthc Policy 2020 13 1271 8 32904130 10.2147/RMHP.S258785 PMC7450521

[63] Kackin O Ciydem E Aci OS Kutlu FY Experiences and psychosocial problems of nurses caring for patients diagnosed with COVID-19 in Turkey: A qualitative study Int J Soc Psychiatry 2021 67 2021 2 158 67 32674644 10.1177/0020764020942788

[64] Galehdar N Kamran A Toulabi T Heydari H Exploring nurses’ experiences of psychological distress during care of patients with COVID-19: a qualitative study BMC psychiatry 2020 20 1 1 9 33023535 10.1186/s12888-020-02898-1 PMC7538040

[65] Aquilia A Grimley K Jacobs B Kosturko M Mansfield J Mathers C Nursing leadership during COVID-19: Enhancing patient, family and workforce experience Patient Exp J 2020 7 2 136 43

[66] Rosser E Westcott L Ali PA Bosanquet J Castro-Sanchez E Dewing J The Need for Visible Nursing Leadership During COVID-19 J Nurs Scholarsh 2020 1 3 0(0) 10.1111/jnu.12587 PMC7361621 32779857

[67] Tang L Zhao X-M Yu X-Y Team management in critical care units for patients with COVID-19: an experience from Hunan Province, China Crit Care 2020 24 304 32505189 10.1186/s13054-020-02921-7 PMC7275843

[68] Purabdollah M Ghasempour M Tele-Nursing New Opportunity for Nursing Care in COVID-19 Pandemic Crisis Iran J Public Health 2020 49 130 1 34268221 10.18502/ijph.v49iS1.3685 PMC8266018

[69] Joo JY Liu MF Nurses’ barriers to caring for patients with COVID-19: a qualitative systematic review Int Nurs Rev 2021 1 12 0 33420749 10.1111/inr.12648 PMC8013562

[70] Morley G Grady C Mccarthy J Ulrich CM Covid-19: Ethical Challenges for Nurses Hastings Cent Rep 2020 50 3 35 9 10.1002/hast.1110 PMC7272859 32410225

[71] Chan PS Berg RA Nadkarni VM Code blue during the COVID-19 pandemic Circ Cardiovasc Qual Outcomes 2020 13 5 e006779 32255661 10.1161/CIRCOUTCOMES.120.006779 PMC7237295

[72] Kirkpatrick JN Hull SC Fedson S Mullen B Goodlin SJ Scarce-resource allocation and patient triage during the COVID-19 pandemic: JACC Review Topic of the Week J Am Coll Cardiol 2020 76 1 85 92 32407772 10.1016/j.jacc.2020.05.006 PMC7213960

[73] Savary D Morin F Fadel M Metton P Richard J Descatha A Considering the challenge of the Covid-19 pandemic, is there a need to adapt the guidelines for basic life support resuscitation? Resuscitation 2020 152 50 32234368 10.1016/j.resuscitation.2020.03.010 PMC7270524

[74] Behrens KG Clinical ethical challenges in the Covid-19 crisis in South Africa Wits J Clin Med 2020 2 SI 29 32

[75] Nishimura M High-flow nasal cannula oxygen therapy in adults J Intensive Care 2015 3 1 15 25866645 10.1186/s40560-015-0084-5 PMC4393594

[76] Nishimura M High-flow nasal cannula oxygen therapy in adults: physiological benefits, indication, clinical benefits, and adverse effects Respir Care 2016 61 4 529 41 27016353 10.4187/respcare.04577

[77] Daly KJ The role of the ECMO specialist nurse Qatar Med J 2017 1 54

[78] Calhoun A ECMO: Nursing Care of Adult Patients on ECMO Crit Care Nurs Q 2018 41 4 394 8 30153183 10.1097/CNQ.0000000000000226

